# An Updated Phylogeny of the Human Y-Chromosome Lineage O2a-M95 with Novel SNPs

**DOI:** 10.1371/journal.pone.0101020

**Published:** 2014-06-27

**Authors:** Xiaoming Zhang, Jatupol Kampuansai, Xuebin Qi, Shi Yan, Zhaohui Yang, Bun Serey, Tuot Sovannary, Long Bunnath, Hong Seang Aun, Ham Samnom, Wibhu Kutanan, Xin Luo, Shiyu Liao, Daoroong Kangwanpong, Li Jin, Hong Shi, Bing Su

**Affiliations:** 1 State Key Laboratory of Genetic Resources and Evolution, Kunming Institute of Zoology, Chinese Academy of Sciences, Kunming, China; 2 Department of Biology, Faculty of Science, Chiang Mai University, Chiang Mai, Thailand; 3 State Key Laboratory of Genetic Engineering and Ministry of Education, Key Laboratory of Contemporary Anthropology, School of Life Sciences and Institutes of Biomedical Sciences, Fudan University, Shanghai, China; 4 Department of Geography and Land Management, Royal University of Phnom Penh, Phnom Penh, Cambodia; 5 Capacity Development Facilitator for Handicap International Federation and Freelance Research, Battambang, Cambodia; 6 Kunming College of Life Science, University of Chinese Academy of Sciences, Beijing, China; 7 Department of Biology, Faculty of Science, Khon Kaen University, Khon Kaen, Thailand; University of New South Wales, Australia

## Abstract

Though the Y-chromosome O2a-M95 lineage is one of the major haplogroups present in eastern Asian populations, especially among Austro-Asiatic speaking populations from Southwestern China and mainland Southeast Asia, to date its phylogeny lacks structure due to only one downstream SNP marker (M88) assigned to the lineage. A recent array-capture-based Y chromosome sequencing of Asian samples has yielded a variety of novel SNPs purportedly belonging to the O2a-M95 lineage, but their phylogenetic positions have yet to be determined. In this study, we sampled 646 unrelated males from 22 Austro-Asiatic speaking populations from Cambodia, Thailand and Southwestern China, and genotyped 12 SNP makers among the sampled populations, including 10 of the newly reported markers. Among the 646 males, 343 belonged to the O2a-M95 lineage, confirming the supposed dominance of this Y chromosome lineage in Austro-Asiatic speaking populations. We further characterized the phylogeny of O2a-M95 by defining 5 sub-branches: O2a1*-M95, O2a1a-F789, O2a1b*-F1252, O2a1b1*-M88 and O2a1b1a -F761. This updated phylogeny not only improves the resolution of this lineage, but also allows for greater tracing of the prehistory of human populations in eastern Asia and the Pacific, which may yield novel insights into the patterns of language diversification and population movement in these regions.

## Introduction

As the global architecture of human Y-chromosome phylogeny has become increasingly well-defined, researchers have found a powerful tool that helps explain a great deal of human population history that was previously inaccessible [1]–[3]. For eastern and southeastern Asia, the Y-chromosome haplogroup O-M175 is particularly important, as it is the most prevalent Y-chromosome lineage in these regions and comprises around 75% of the male populations in mainland China [4]–[7] and roughly 87% in Southeast Asia [8]–[13]. To date, studies have shown three major sub-lineages under O-M175: O1a-M119, O2a-M95 and O3-M122 [14]. The extant phylogenies of O3-M122 and O1a-M119 have been adequately resolved with many SNP markers, and subsequently studied in many Asian populations [4], [10], [14]. However, O2a-M95, which comprises some 58% of the male populations in Southeast Asia [8]–[13], [15], the phylogeny still lacks resolution, with only two characterized sub-branches (O2a1*-M95 and O2a1a-M88) [16], greatly limiting the genetic and historical inferences that can be made from this key Y chromosome lineage in Asia and the Pacific.

The importance of O2a-M95, aside from its genetic prevalence, is its predominance among populations of the Austro-Asiatic language family, the eighth largest family in the world in terms of population size (104 millions) [17]. In Southeast Asia, Austro-Asiatic is the first language of many ethnic groups in Cambodia, Vietnam, Laos, Thailand, Burma and Malaysia, and serves as the main official language in Cambodia and Vietnam. More importantly, a recent genome-wide survey of sequence variations in extensive Asian populations found that the Austro-Asiatic speaking populations are located at the basal position of the phylogenetic tree covering all major Asian populations, suggesting that they may represent one of the most ancient populations in Southeast Asia [18]. We recently demonstrated that the Austro-Asiatic speaking populations from Cambodia harbor many ancient polymorphisms in their mitochondrial genomes, consistent with the proposed ancientness [19]. The postulated southern origin and northward migration of East Asian populations then places mainland Southeast Asia (MSEA) and southern China as the potential cradle of modern human settlement during their initial dispersal into eastern Asia [4], [5], [15], [18], [20]. Though a variety of data supports this position, this theory needs greater evidence to more accurately trace the history of early human migration into Asia. As the major Y chromosome lineage in Austro-Asiatic populations, improving the phylogenetic resolution of O2a-M95 would greatly improve our understanding of early human migrations in Asia and the Pacific.

In this study, we aimed to improve the resolution of the O2a-M95 lineage by analyzing the newly discovered SNP markers among Austro-Asiatic speaking populations. After genotyping of 10 novel Y chromosome SNPs in 22 Austro-Asiatic populations from Cambodia, Thailand and southwestern China, we were able to markedly improve the resolution of O2a-M95 and establish 5 new sub-branches, providing a more detailed within-lineage structure for this key Y chromosome lineage.

## Materials and Methods

**Figure 1:**
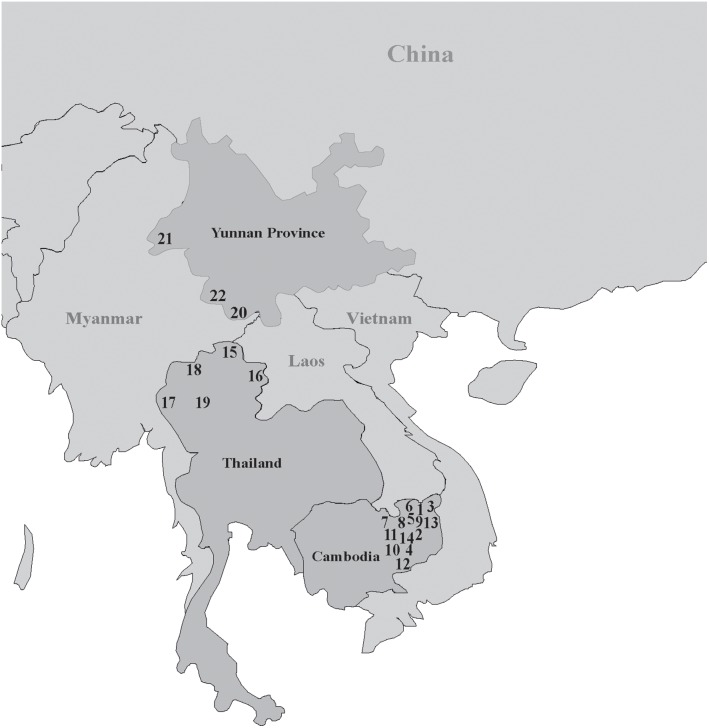
Geographic locations of 22 populations sampled in present study. The numbers refer to the populations, corresponding to the order of populations in Figure 2.

To dissect the phylogeny of the O2a-M95 lineage, we collected blood samples from 646 unrelated male individuals from Cambodia, Thailand and southwestern China (Yunnan province) who belong to 22 ethnic populations (Figure 1). Aside for Jarai and Lao, who belong to the Austronesian and Daic language family respectively, all the other sampled males were from Austro-Asiatic speaking populations.

We first genotyped M95 in all samples using Sanger sequencing (rs2032650, Y-position 20397832, amplicon length: 480 bp). For those samples possessing the derived alleles at M95 (343 in total), using the Snapshot method described previously [21], we next genotyped 10 novel SNPs (F2176, F987, F1252, F789, F4181, F2346, F761, F2758, F2411 and F1399; descriptions in Table 1). These 10 SNPs were reported having derived alleles at M95 (M95^der^), but had no clear phylogenetic positions due to the limited sample size in the previous study [22]. Additionally, PK4 and M88 were also genotyped for all M95^der^ samples with Snapshot. Written informed consent was obtained from all subjects prior to any study-related procedures. The research protocols of this study were approved by the internal review board of Kunming Institute of Zoology, Chinese Academy of Sciences and adhered to all the relevant national and international regulations.

**Table 1:** Description of 10 novel SNPs genotyped in 343 M95^der^ males in this study.

SNP	Y-position(hg19)	Ref	Mut	ForwardPrimer*	ReversePrimer*	PCRlength (bp)	SNPposition#
F2176	16477410	G	A	AGCAGGTAAGGATCAATAGG	AGTCACTCAGAATAGCAACT	425	211
F987	7543143	A	T	CTTTCTTTCTTGTGAGTCTGTC	ATGCGAGTGTAGTTGGAAG	271	140
F1252	8492876	C	T	GCTGTCTGAATCTCTACCAT	GGCATGACTAAGGCATCC	377	247
F789	6629330	T	A	CACTGTTGCTGCTCCATT	CATCTTCCTGAATATCTGTCTG	407	181
F4181	15043403	A	G	GATGCCTTCAGATACTTAGC	CTCTCAGTCCTCATTGTCAT	186	134
F2346	16967534	C	T	CCTCATAAGAGCCATTACTTC	ACACATCCTTAGCCATACAT	488	248
F761	6136156	A	G	GGTAGTGGAAGGAAGATGAT	AGAAGTTAAGGCTGCTGTT	170	100
F2758	18415345	C	T	CTGCTAGTAGACTATTGAAGAC	GTAAGGCATCACCTGTCA	374	225
F2411	17184198	C	G	GCTTGTCACTCAATTCTTCA	ACCTTGTAGTGTAGCATCAT	371	237
F1399	8762969	C	A	ACCAACTCAACCTCATACTC	CACTTGACCGAAGACCTAG	234	66

*Designed in this study;

# The precise position of the variable nucleotide in the amplicon.

## Results

**Figure 2:**
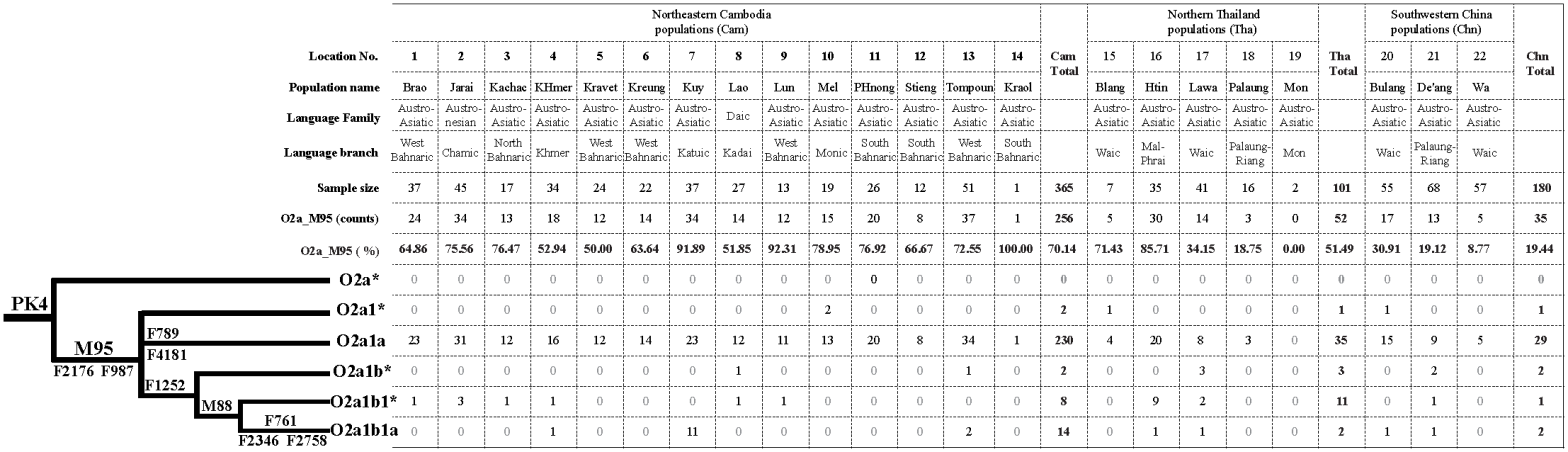
Updated phylogenetic tree of the human Y-chromosome lineage O2a-M95.

Among the 646 male individuals, 343 of them (53.10%) belong to the O2a-M95 lineage, consistent with previous studies of Southeast Asian populations [8]–[13]. For these M95^der^ individuals, we genotyped 10 novel SNPs as well as PK4 and M88 (genotyping results are shown in Table S1) and the results allowed us to update the phylogenetic tree of O2a-M95 (Figure 2). The parsimony rule was applied for tree construction. For example, both F2176 and F987 showed derived status in all M95^der^ samples, supporting that they are equivalent with M95 in the phylogenetic tree (Figure 2). For F1252, some individuals showed derived and some showed ancestral status, indicating that F1252 is a downstream SNP of M95 (Figure 2). It should be noted that both F2411 and F1399 showed ancestral status in all M95^der^ samples, suggesting that they do not belong to the O2a-M95 lineage, and previous phylogenetic positions of these two SNPs were not correctly allocated [22].

In the updated phylogenetic tree, we established 5 sub-branches: O2a1*-M95 (other equivalent SNPs are F2176 and F987; 4/343), O2a1a-F789 (the other equivalent SNP is F4181; 294/343), O2a1b*-F1252 (7/343), O2a1b1*-M88 (20/343) and O2a1b1a -F761 (other equivalent SNPs are F2346 and F758; 18/343) (Figure 2). Among the 5 newly defined sub-branches, O2a1a-F789 was the most frequent branch, accounting for 85.71% of all the samples. PK4 was first reported in a population in Pakistan [23], and later in both Nepal and India [24]. Previously, PK4 was assigned as a downstream marker of M88 [14]. However, after further scanning, samples possessing PK4^der^/M95^anc^/M88^anc^ genotypes were identified and PK4 was placed upstream of M95 [16]. In this study, all M95^der^ samples are also PK4^der^, consistent with the recent update of PK4 [16].

The previous O2a1a-M88 sub-branch [16] was further split into two sub-branches, named O2a1b1*-M88, and O2a1b1a-F761. Totally, 3 of the 10 novel SNPs (F761, F2346 and F2758) were assigned to the sub-branch O2a1b1a-F761 (Figure 2). F1252 turned out to be an upstream maker of M88, parallel with the previous O2a1*-M95 lineage and a newly defined O2a1a-F789 lineage (defined by F789 and F4181) (Figure 2). However, F2411 and F1399 were not polymorphic and showed ancestral alleles for M95 in all tested samples (Supplementary Table 1), and as such these two SNPs do not belong to the O2a-M95 lineage, suggesting an earlier misplaced phylogenetic position of these markers under M95 [22].

The geographic distributions of the 5 newly defined M95 sub-lineages are similar among regional populations from Southwestern China (Yunnan province), Thailand and Cambodia, with O2ala-F789 being the most frequent sub-lineage in all three regional populations (89.84% in Cambodia; 67.31% in Thailand and 82.86% in Yunnan), followed by O2a1b-F1252 (9.38% in Cambodia, 30.77% in Thailand and 14.29% in Yunnan). The other three sub-lineages are relatively rare. Notably, two of the sub-lineages have unusually high frequencies in Kuy (O2a1b1a) and Htin (O2a1b1), both of which belong to distinct language branches of the Austro-Asiatic family. Whether this ethnic-specific pattern reflects a unique population history or just a sampling bias need to be tested in the future.

## Discussion

The genotyping of 10 novel SNPs as well as PK4 and M88 found in 22 Austro-Asiatic speaking ethnic populations from Southwestern China and Southeast Asia allowed us to greatly enhance and update the existing phylogeny of the Y-chromosome O2a-M95 lineage with much greater resolution. The final results show that the O2a-M95 lineage is the most predominant Y chromosome lineage in Southeast Asia, and can be divided into 5 sub-branches. This more enhanced view should be extremely useful in further follow-up studies aimed at piecing together the currently fragmented population histories in Asia and the Pacific.

Due to the predominant presence of Austro-Asiatic speaking populations from Southeast Asia as well as the historical record of these populations movements and activities, O2a-M95 has also been shown to be prevalent in other populations from the southern part of Asia, such as the Austro-Asiatic speaking populations in India (average 84.66%) [25], [26], the Daic and Hmong-Mien speaking populations in China (average 45.25%) [8], [10], [27]–[29], the Austronesian speaking populations (about 27.90%) in Island Southeast Asia (including Taiwan aborigines) [10]–[12], [26]. Given this broad dispersal, exploring the origin and movement of O2a-M95 across the region is not only informative for tracing prehistoric migrations, but also for understanding the origin and diversification of language families in Asia and clarifying many details of the region’s history that have remained, till now, unclear at best.

## Supporting Information

Table S1 Genotyping results of the M95^der^ samples.
(XLSX)
